# Construction Notes

**Published:** 1894-04-28

**Authors:** 


### CONSTRUCTION NOTES.

**Ashby-de-la-Zouch Cottage Hospital.**

After many months of labour on the part of a number of ladies and gentlemen of the town, a cottage hospital for the use of the town and district has just been opened. Lady Cave performed the opening ceremony. The hospital gives accommodation for six adults and three children, is amply furnished, and forms a picture of neatness and orderliness. It is on the property of Lord Donington, but was obtained on a lease by the organisers, who urged the necessity for such accommodation to Ashby, and hope to make progress and build eventually a permanent hospital.

